# Mutated NPM1 in combination with overexpression of Meis1 or Hoxa9 is not sufficient to induce acute myeloid leukemia

**DOI:** 10.1186/s40164-016-0053-2

**Published:** 2016-08-11

**Authors:** Hanna Grauers Wiktorin, Tina Nilsson, Ann Jansson, Lars Palmqvist, Anna Martner

**Affiliations:** 1 Sahlgrenska Cancer Center, Sahlgrenska Academy, University of Gothenburg, Gothenburg, Sweden; 2 Department of Clinical Chemistry, Sahlgrenska University Hospital, Bruna Stråket 16, 413 45 Gothenburg, Sweden; 3 Department of Clinical Chemistry and Transfusion Medicine, Institute of Biomedicine, Sahlgrenska Academy, University of Gothenburg, Gothenburg, Sweden

**Keywords:** Acute myeloid leukemia, NPM1, Meis1, Hoxa9

## Abstract

**Background:**

Acute myeloid leukemia (AML) carrying nucleophosmin 1 (NPM1) mutations (NPMc^+^) is regarded as a separate entity of myeloid neoplasms due to its distinct biological and clinical features. However, NPMc^+^ alone displays low leukemogenic activity and cooperating events appear crucial for AML to develop. Dysregulation of homeobox genes, such as HOXA9 and MEIS1, is a common transcriptional signature of NPMc^+^ AML. Furthermore, the pathogenic role for NPMc^+^ in AML remains incompletely understood.

**Aim:**

To elucidate if NPMc^+^ collaborates with Meis1 or Hoxa9 in the evolvement of AML.

**Methods:**

Murine bone marrow cells were genetically engineered to express mutated NPM1 variant A in combination with overexpression of Meis1 or Hoxa9. The capacity of the transduced cells to transform in vitro and to cause leukemia in vivo was then assessed.

**Findings and conclusion:**

There was no synergy between NPMc^+^ and Meis1 or Hoxa9 in causing leukemogenic transformation of murine bone marrow cells, or in inducing AML in a transplantation model. Hence, overexpression of Meis1 or Hoxa9 in combination with NPMc^+^ expression was not sufficient to generate an NPMc^+^ AML mouse model.

## Background

Nucleophosmin (NPM1) is found mutated in about 30 % of acute myeloid leukemia (AML) cases, making it one of the most commonly mutated genes in AML [1, 2]. Wild-type NPM1 has been ascribed many biological functions, including a role in the biogenesis of ribosomes, genomic stability and transport of small basic proteins to the nucleus [3]. The NPM1 mutations occurring in AML disrupts the nucleolar localization signal of NPM1 and generates a nuclear export signal in its place, resulting in aberrant cytoplasmic localization of mutated NPM1 (NPMc^+^) [4]. Since 2008, the World Health Organization regards AML with mutated NPM1 as a provisional AML entity due to its unique characteristics [5]. Despite the recognition of mutated NPM1 as a founder genetic lesion [6] and a putative target for novel therapy [7], the mechanism by which NPMc^+^ contributes to leukemogenesis has only partly been explored. Animal models of NPMc^+^ AML, aiming at understanding the NPM1-driven leukemogenesis, have thus far revealed that NPMc^+^ alone displays low leukemogenic activity [8, 9] and that additional cooperative mutations, such as FLT3-ITD [10] or N-ras mutations [9] are required for AML to develop.

Gene expression data from both NPM1-mutated adult and pediatric AML cases have identified an association between NPMc^+^ and dysregulated expression of homeobox (HOX) and TALE genes, including Hoxa9 and Meis1, which are known to be involved in hematopoietic development [11–14]. In accordance, Ogawara et al. reported that NPMc^+^ expression in c-Kit^+^ bone marrow (BM) cells results in increased expression of Hoxa9 [15]. Hoxa9 and Meis1 are primarily expressed at the level of hematopoietic stem and progenitor cells during normal hematopoiesis [16] and have been shown to cooperate in causing AML in transplantation mouse models [17, 18]. The presumed NPMc^+^-induced Hoxa9 expression incited us to investigate a potential synergy between NPMc^+^ and Meis1 in causing AML. To test this hypothesis, and in an attempt to develop a transplantation model reliant on NPMc^+^ expression for in vivo onset of AML, C57BL/6 J bone marrow cells were transduced to express NPMc^+^ in combination with overexpression of either Meis1 or Hoxa9. However, we could not find any evidence for cooperation between NPMc^+^ expression and Meis1 or Hoxa9 overexpression in leukemogenic transformation. Thus, our data show that overexpression of Meis1 or Hoxa9 is not sufficient to cause AML in combination with NPMc^+^ in a murine bone marrow transplantation model.

## Methods

### Retroviral vectors

The following vectors have been previously described: MSCV-IRES-GFP (GFP virus) [19], MSCV-IRES-YFP (YFP virus) [20], MSCV-IRES-neo (neo virus) [21], MSCV-HA-Meis1a-IRES-YFP (Meis1 virus) [19], and MSCV-Hoxa9-IRES-neo (Hoxa9 virus) [21]. The sequence for the most common NPM1 mutation, mutation A, was purchased from Integrated DNA Technologies (Coralville, IA, USA), inserted into the MSCV-IRES-GFP vector and used to transiently transfect Phoenix Eco cells (ATCC, LGC Standards GmbH, Wesel, Germany). Virus-containing medium from Phoenix Eco was utilized to transduce GP + E86 cells (ATCC, LGC Standards GmbH, Wesel, Germany) and generate a stable NPMc^+^ viral producer.

### Transduction of C57BL/6 J bone marrow cells

All mice experiments were approved by the Animal Ethics Research Committee in Gothenburg. C57BL/6 J mice were obtained from Charles River Laboratories (Sulzfeld, Germany). Transduction of C57BL/6 J BM with viral vectors was performed as previously described [22]. In brief, murine BM cells harvested from C57BL/6 J mice 4 days after 150 µg/g 5-fluorouracil (5-FU, Accord Healthcare AB, Solna, Sweden) treatment were transduced with NPMc^+^ and neo or YFP viruses (NPMc^+^ cells), Meis1 and GFP viruses (Meis1 cells), Hoxa9 and GFP viruses (Hoxa9 cells), Meis1 and NPMc^+^ viruses (Meis1-NPMc^+^ cells) or Hoxa9 and NPMc^+^ viruses (Hoxa9-NPMc^+^ cells) and cultured in Dulbecco modified Eagle medium (DMEM with high glucose, D6429, Sigma-Aldrich Sweden AB, Stockholm, Sweden) supplemented with 15 % fetal bovine serum (6250, StemCell Technologies Inc., Vancouver, Canada), 2 mM l-glutamine (G7513-100 ml, Sigma-Aldrich Sweden AB, Stockholm, Sweden), 1 % Penicillin and streptomycin (P4333-100 ml, Sigma-Aldrich Sweden AB, Stockholm, Sweden), 10 ng/ml human interleukin-6 (2506, StemCell Technologies SARL, Grenoble, France), 6 ng/ml murine interleukin-3 (2733, StemCell Technologies SARL, Grenoble, France), and 50 ng/ml murine stem cell factor (2931 StemCell Technologies SARL, Grenoble, France) (complete medium). Transduction was achieved by co-culturing 5-FU treated murine BM cells with irradiated (2 × 25 Gray) GP + E86 viral cells in complete medium supplemented with 5 µg/ml protamine sulfate (P-4020, Sigma-Aldrich Sweden AB, Stockholm, Sweden). Two days later, gentle flushing of the wells separated non-adherent BM cells from adherent viral cells. BM cells were pelleted, resuspended in fresh complete culture medium and expanded for 5 days. Selection of transduced cells was achieved by addition of 0.3 mg/ml G418 disulfate salt solution (G8168-10 ml, Sigma-Aldrich Sweden AB, Stockholm, Sweden) to the culture medium followed by sorting of GFP^+^/YFP^+^ cells on a three-laser BD FACSAria (405, 488 and 633 nm from BD Biosciences).

### BM transplantation of C57BL/6 J mice

Transduced BM cells were injected into the tail vein of lethally irradiated (800 cGray) C57BL/6 J mice 1 day after FACS selection of transduced cells. Transplants consisted of 100 000 to 200 000 NPMc^+^, Meis1, Hoxa9, Meis1-NPMc^+^ or Hoxa9-NPMc^+^ cells and two million life-sparing naïve BM cells. Mice were monitored daily during the initial 2 weeks for signs of morbidity. Analyzing the percentage of GFP^+^/YFP^+^ cells in peripheral blood, with a four-laser BDLSRFortessa (405, 488, 532 and 640 nm from BD Biosciences), allowed monitoring of engraftment and disease progression in real-time.

### Quantitative real-time PCR

BM cells were stored in RNAprotect Cell Reagent (76526, Qiagen AB, Sollentuna, Sweden) until total RNA was extracted using the RNeasy Plus Mini kit (74136, Qiagen AB, Sollentuna, Sweden) and cDNA synthesized by SuperScript III First-Strand Synthesis SuperMix for qRT-PCR (11752, Invitrogen, Life Technologies Europe BV, Stockholm, Sweden) all according to the manufacturer’s instructions. Gene expression was analyzed by TaqMan Gene Expression Assays; hNPM1, Hs02339479_g1; hNPM1mutA, Hs00000953_mu; mHoxa9, Mm00439364_m1; hMeis1, Hs00180020_m1; and as reference gene, mHPRT1, Mm01545399_m1 (Applied Biosystems, Life Technologies Europe BV, Stockholm, Sweden) according to the manufacturer’s instructions on a ABI PRISM^®^7900HT instrument (Applied Biosystems, Life Technologies Europe BV, Stockholm, Sweden). Gene expression results are presented as 2^-[Ct(target)-Ct (HPRT1)].

### Sequencing

Genomic DNA was isolated from the BM cells 21 days post transduction with the DNeasy Blood & Tissue kit (69504, Qiagen AB, Sollentuna, Sweden). The sequence over the NPM1 mutation site was analyzed by Sanger sequencing utilizing the BigDye Terminator v3.1 Cycle Sequencing kit (4337455, Applied Biosystems, ThermoFisher Scientific, Hagersten, Sweden) on a 3130xl Genetic Analyzer.

### Methylcellulose colony forming assay

Colony forming unit (CFU) assay was used to evaluate the proliferative capacity of cells after transduction with above mentioned genes. 100 to 5000 cells were seeded in methylcellulose medium (Methocult M3434, StemCell Technologies SARL, Grenoble, France). After 7 days, the number of colonies was counted and the following day cells were replated.

### May-Grünwald-Giemsa staining

Using a Shandon CytoSpin 2 (Axel Johnson Instrument AB, Stockholm, Sweden), transduced BM cells were sprayed onto glass slides and allowed to air-dry. Slides were May-Grünwald-Giemsa stained and images taken on a Nikon Eclipse 90*i* microscope.

### Statistics

For statistical analyses, Student´s unpaired *t* test was performed. All statistical analyses were calculated using GraphPad Prism Version 6.0.

## Results

**Fig. 1:**
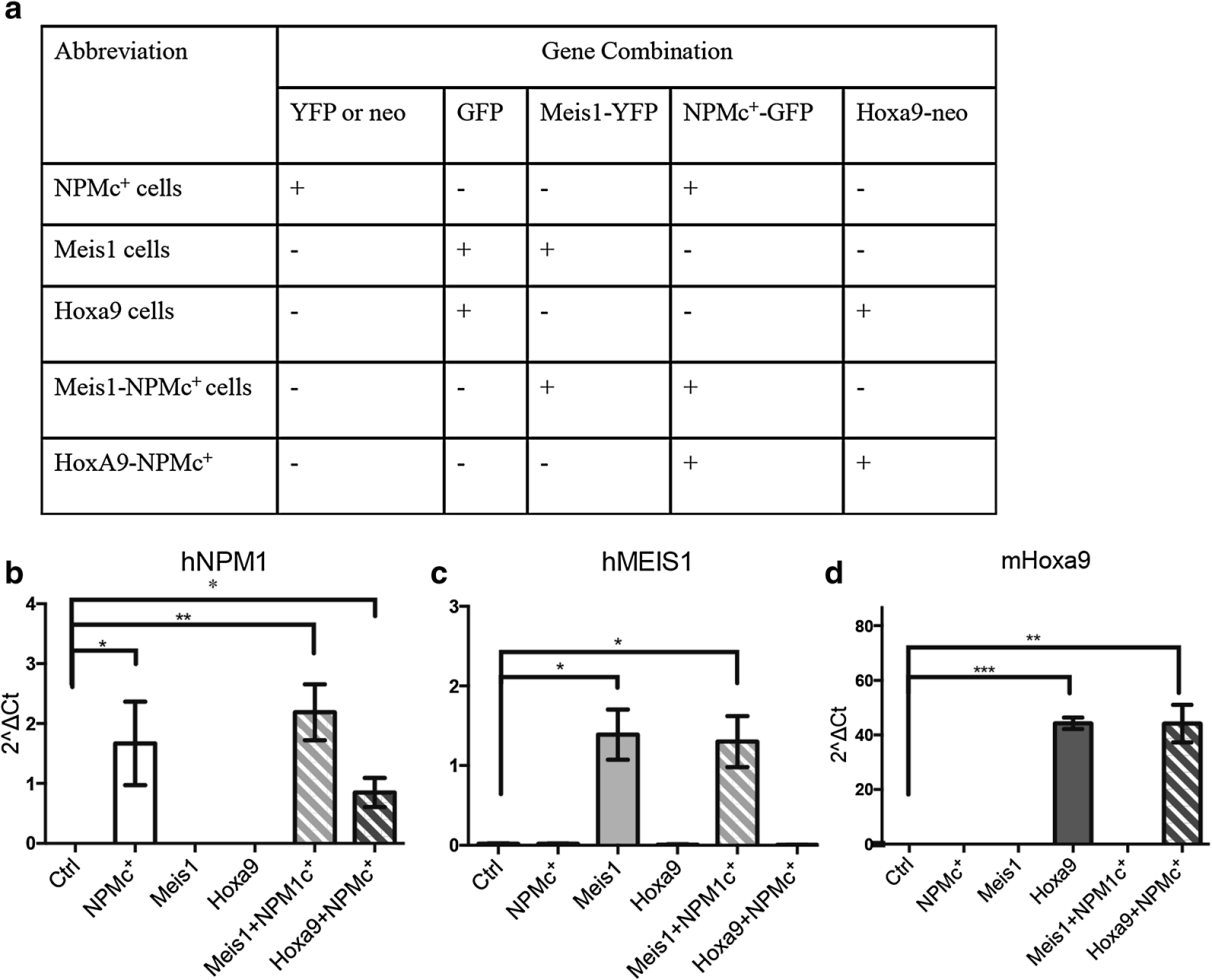
Transduction of murine bone marrow cells with NPMc^+^, Meis1 and Hoxa9. **a** The table shows the combination of genes murine bone marrow cells were transduced with. **b**–**d** RNA was extracted from transduced cells using the RNeasy Plus Mini kit and analyzed by qPCR for expression of human NPM1 (**b**), human MEIS1 (**c**) and murine Hoxa9 (**d**). For all qPCR analysis n = 3. The bars indicate mean ± SD. Statistical calculations were performed using Student´s unpaired t test. *p < 0.05, **p < 0.01, ***p < 0.001

To clarify whether NPMc^+^ and Meis1 or Hoxa9 cooperate in induction of AML, 5-FU-treated BM cells from C57BL/6 J mice were transduced with aforementioned genes as outlined in Fig. 1a. Successfully transduced cells were FACS-sorted based on GFP and/or YFP expression and sorted cells were utilized in the further experiments. Quantitative PCR (qPCR) analysis confirmed increased mRNA expression levels of NPMc^+^, Meis1 and Hoxa9 in all transduced cells (Fig. 1b–d). The presence of the correct sequence of mutated NPM1 in the transduced NPMc^+^ BM cells was verified by sequencing. No enhancement of Hoxa9 expression was observed in the NPMc^+^ liquid cultures (Fig. 1d).

**Fig. 2:**
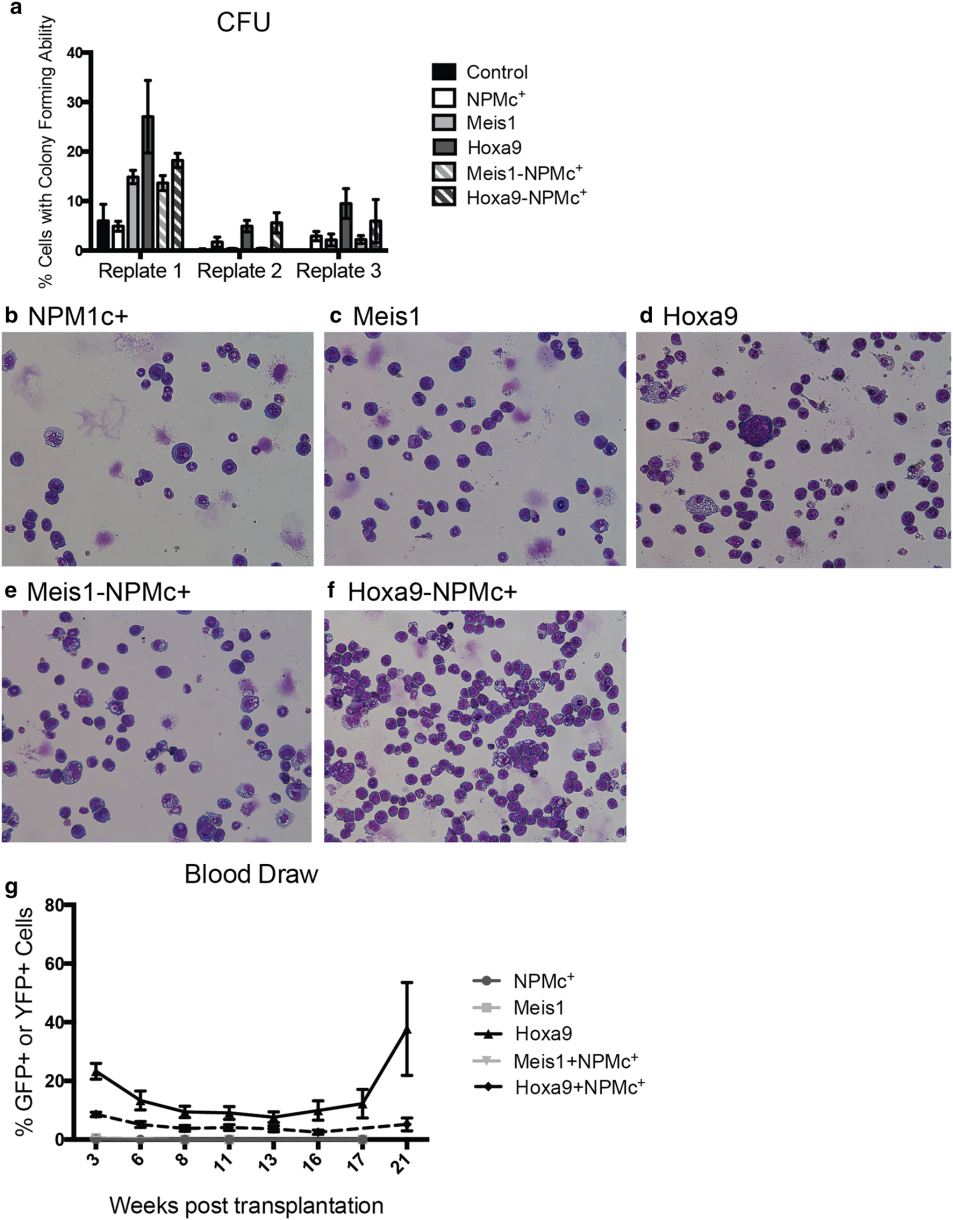
No synergy in transforming capacity of NPMc^+^ and Meis1 or Hoxa9. **a** Percentage of single and double transduced cells forming colonies on methocult plates after serial replatings: Control (empty GFP+/YFP+ vector) (n = 4 at replate 1 and n = 2 at replate 2), NPMc+ (n = 7 at replate 1 and 2 and n = 5 at replate 3), Meis1 (n = 3), Hoxa9 (n = 10 at replate 1 and 2, n = 7 at replate 3), Meis1-NPMc^+^ (n = 3), and Hoxa9-NPMc^+^ (n = 4 at replate 1 and 2 and n = 2 at replate 3). **b**–**f** Representative images from May-Grünewald-Giemsa staining showing NPMc^+^ cells (**b**), Meis1 cells (**c**), Hoxa9 cells (**d**), Meis1-NPMc^+^ cells (**e**) and Hoxa9-NPMc^+^ cells (**f**). **g** The percentage of NPMc^+^ , Meis1, Hoxa9, Meis1-NPMc^+^ and Hoxa9-NPMc^+^ cells (reflected by GFP+/YFP+ expression) in peripheral blood was monitored by flow cytometry during 21 weeks. NPMc^+^ (n = 8), Meis1 (n = 5), Hoxa9 cells (n = 5), Meis1-NPMc^+^ cells (n = 5), and Hoxa9-NPMc^+^ (n = 10). *Error bars* show ± standard error of the mean (SEM)

Next, the transfected cells were analyzed by CFU assays to investigate a potential synergy between NPMc^+^ and Meis1 or Hoxa9 in leukemogenic transformation. Cells were cultured in methylcellulose medium for 7 days before enumeration of colonies. Cells transduced with NPMc^+^, Meis1 or Hoxa9 all showed enhanced serial colony-forming activity, compared with control cells transduced only with the selection markers, neo, GFP or YFP, where virtually no colonies were formed after the first replating (Fig. 2a). The colony forming capacity was highest in cells overexpressing Hoxa9 but no significant differences were observed in colony formation of single transduced cells and cells transduced to express both NPMc^+^ and Meis1 or NPMc^+^ and Hoxa9 (Fig. 2a). Similarly, it was noticed that only cells overexpressing Hoxa9 (in the presence or absence of NPMc^+^) became immortalized and survived long-term in vitro cultures.

In support of the necessity of increased Hoxa9 levels for improved proliferative capacity, May-Grünewald-Giemsa staining of cells revealed that Hoxa9 expression was required for cells to maintain a blast-like cell-morphology (Fig. 2d and f). In the absence of increased Hoxa9 expression levels, cells differentiated and mainly displayed the morphology of monocytes, macrophages and neutrophils (Fig. 2b, c and e).

Colony-forming capability in vitro often mirrors the potential of cells to engraft in mice [23]. In accordance, transplantation with NPMc^+^, Meis1 or Meis1-NPMc^+^ cells (in combination with life-sparing BM cells) to lethally irradiated C57BL/6 J mice did not lead to long-term engraftment, as the percentage of transfected cells in the blood of the mice was consistently below 1.5 % (Fig. 2g). In contrast, transplantation with Hoxa9 and Hoxa9-NPMc^+^ cells led to long-term engraftment of leukemic cells, albeit at a low level (Fig. 2g). Three out of five mice transplanted with Hoxa9 cells developed late onset leukemia, with the first mice progressing into leukemia displaying disease symptoms, as determined by increased white blood cell counts along with an increased frequency of GFP^+^/YFP^+^ leukemic cells in blood, approximately 16 weeks after transplantation (Fig. 2g). None of the mice transplanted with Hoxa9-NPMc^+^ cells showed signs of disease progression within the 20-week observation period (Fig. 2g).

## Discussion

Our in vitro and in vivo results consistently demonstrate a lack of synergy between NPMc^+^ and Meis1 or Hoxa9 in terms of transforming murine bone marrow cells and causing AML in an experimental transplantation model. Although NPMc^+^ has been associated with enhanced Hoxa9 expression in AML patients [11] and in transduced murine BM cells [15], we did not observe enhanced expression in our NPMc^+^ liquid cultures (Fig. 1d). The reason for absent Hoxa9 induction and for the low leukemogenic capacity of the NPMc^+^ cells utilized in our study might be that normal levels of wild-type NPM1 was expressed by the transduced BM cells. Hence, *NPM1* mutations in patients might cause disease both by causing a reduced expression of wild-type NPM1 (expression from only one allele), and by the oncogenic activity of NPMc^+^. *NPM1* mutations are always heterozygous and a complete knock out of the protein results in embryonic lethality in mice [24]. However, mice genetically engineered to express only one wild-type NPM1 allele display a higher susceptibility to hematological malignancies, including myeloid leukemia, indicating that loss of NPM1 function is a mechanism of pathogenicity [25].

Since *NPM1* mutations in AML always result in cytoplasmic localization of the protein, it is conceivable that NPMc^+^ plays a crucial role in pathogenicity. NPMc^+^ has been ascribed oncogenic functions [26–28] and transgenic mice expressing NPMc^+^ within the myeloid compartment develop a myeloproliferative disease, albeit no AML [8]. To mimic the situation in human AML, conditional knock-in models, referred to as *Npm1* ^*cA/*+^ mice, have been developed where NPMc^+^ is expressed in mice that display reduced expression-levels of wild-type NPM1. In this setting, one-third of the *Npm1* ^*cA/*+^ mice developed late onset AML, indicating a more aggressive course of disease [9].

Furthermore, when NPMc^+^ was introduced to BM cells from transgenic NPM1^+/−^ mice, the increase in Hoxa9 expression was much greater than when NPMc^+^ was introduced to wild type BM cells [15]. However, in this previous study, even NPMc^+^ expressing NPM^+/−^ bone marrow cells failed to cause AML when transplanted into irradiated mice, highlighting the importance of cooperative genes [15]. Thus, even in conditions of reduced wild type NPM1 expression, the selection of genes to cooperate with NPMc^+^ in triggering AML is crucial. We and others have previously shown that Hoxa9 and Meis1 act in synergy to cause leukemia in transplantation models [17, 29]. Since NPMc^+^ should trigger an enhanced Hoxa9 expression on a NPM1^+/−^ background [15], it is conceivable that NPMc^+^ should cooperates with Meis1 in causing AML if NPM1^+/−^ bone marrow cells would be utilized, which merits further studies. Cells overexpressing Hoxa9 showed an increased colony forming and disease causing capacity compared with cells not overexpressing Hoxa9. However, our results indicate, if anything, a reduced disease causing potential of Hoxa9-NPMc^+^ cells compared with Hoxa9 overexpressing cells alone, which do not incite additional studies of synergy between NPMc^+^ and Hoxa9.

## Abbreviations

AML: acute myeloid leukemia
NPM1: nucleophosmin 1
NPMc^+^: cytoplasmic NPM1
HOX: homeobox
CFU: colony forming unit
BM: bone marrow
